# Association of Oral Corticosteroid Bursts With Severe Adverse Events in Children

**DOI:** 10.1001/jamapediatrics.2021.0433

**Published:** 2021-04-19

**Authors:** Tsung-Chieh Yao, Jiu-Yao Wang, Sheng-Mao Chang, Yen-Chen Chang, Yi-Fen Tsai, Ann Chen Wu, Jing-Long Huang, Hui-Ju Tsai

**Affiliations:** 1 Division of Allergy, Asthma, and Rheumatology, Department of Pediatrics, Chang Gung Memorial Hospital, Taoyuan, Taiwan; 2 School of Medicine, Chang Gung University College of Medicine, Taoyuan, Taiwan; 3 Center for Allergy and Clinical Immunology Research, College of Medicine, National Cheng Kung University, Tainan, Taiwan; 4 Department of Pediatrics, National Cheng Kung University Hospital, Tainan, Taiwan; 5 Department of Statistics, National Cheng Kung University, Tainan, Taiwan; 6 Institute of Population Health Sciences, National Health Research Institutes, Zhunan, Taiwan; 7 Precision Medicine and Translational Research Center, Department of Population Medicine, Harvard Pilgrim Health Care Institute and Harvard Medical School, Boston, Massachusetts; 8 Department of Pediatrics, Children’s Hospital, Boston, Massachusetts; 9 Department of Pediatrics, New Taipei Municipal Tu Cheng Hospital, Chang Gung Memorial Hospital, New Taipei, Taiwan

## Abstract

**Importance:**

The adverse effects from the long-term use of oral corticosteroids are known, but, to our knowledge, few studies have reported the risk of corticosteroid bursts, particularly among children.

**Objective:**

To quantify the associations of corticosteroid bursts with severe adverse events, including gastrointestinal (GI) bleeding, sepsis, pneumonia, and glaucoma, in children.

**Design, Setting, and Participants:**

This study used data derived from the National Health Insurance Research Database in Taiwan from January 1, 2013, to December 31, 2017, on children younger than 18 years of age and used a self-controlled case series design. Data were analyzed from January 1 to July 30, 2020.

**Exposure:**

Oral corticosteroid bursts (defined as oral corticosteroid use for ≤14 days).

**Main Outcomes and Measures:**

Incidence rates were calculated of 4 severe adverse events (GI bleeding, sepsis, pneumonia, and glaucoma) in children who did or did not receive corticosteroid bursts. Conditional fixed-effect Poisson regression was used to estimate incidence rate ratios (IRRs) of severe adverse events within 5 to 30 days and 31 to 90 days after initiation of corticosteroid bursts.

**Results:**

Among 4 542 623 children, 23% (1 064 587; 544 268 boys [51.1%]; mean [SD] age, 9.7 [5.8] years) were prescribed a single corticosteroid burst. The most common indications were acute respiratory tract infections and allergic diseases. The incidence rate differences per 1000 person-years between children administered a single corticosteroid burst and those not prescribed corticosteroids were 0.60 (95% CI, 0.55-0.64) for GI bleeding, 0.03 (95% CI, 0.02-0.05) for sepsis, 9.35 (95% CI, 9.19-9.51) for pneumonia, and 0.01 (95% CI, 0.01-0.03) for glaucoma. The IRRs within 5 to 30 days after initiating corticosteroid bursts were 1.41 (95% CI, 1.27-1.57) for GI bleeding, 2.02 (95% CI, 1.55-2.64) for sepsis, 2.19 (95% CI, 2.13-2.25) for pneumonia, and 0.98 (95% CI, 0.85-1.13) for glaucoma; the IRRs within the subsequent 31 to 90 days were 1.10 (95% CI, 1.02-1.19) for GI bleeding, 1.08 (95% CI, 0.88-1.32) for sepsis, 1.09 (95% CI, 1.07-1.11) for pneumonia, and 0.95 (95% CI, 0.85-1.06) for glaucoma.

**Conclusions and Relevance:**

This study suggests that corticosteroid bursts, which are commonly prescribed for children with respiratory and allergic conditions, are associated with a 1.4- to 2.2-fold increased risk of GI bleeding, sepsis, and pneumonia within the first month after initiation of corticosteroid therapy that is attenuated during the subsequent 31 to 90 days.

## Key Points

**Question:**

Are there potential harms associated with oral corticosteroid bursts (defined as the use of oral corticosteroids for 14 or fewer days) in children?

**Findings:**

In this nationwide population-based study of 1 064 587 children who received a single corticosteroid burst, a burst was associated with 1.4- to 2.2-fold increased risk of gastrointestinal bleeding, sepsis, and pneumonia within the first month after corticosteroid initiation.

**Meaning:**

This study suggests that clinicians should be aware of potentially severe adverse events associated with corticosteroid bursts in children.

## Teaser

This study examines the associations of corticosteroid bursts with severe adverse events, including gastrointestinal bleeding, sepsis, pneumonia, and glaucoma, in children.

## Introduction

Oral corticosteroids are the bedrock of treatment for several inflammatory diseases, such as rheumatoid arthritis, inflammatory bowel disease, and asthma, as recommended by international guidelines.^1,2^ It has been well recognized for more than a half century that long-term use of oral corticosteroids is associated with subsequent adverse events, including Cushingoid features, gastrointestinal (GI) bleeding, infections, glaucoma, hyperglycemia, cardiovascular diseases, and osteoporosis.^3,4,5,6,7,8^ Clinicians therefore caution against long-term use of oral corticosteroids unless the potential benefits outweigh the potential risks.

To our knowledge, scant data are available about the potential harms of corticosteroid bursts, which are defined as courses of oral corticosteroids for 14 or fewer days.^9,10,11,12,13,14,15,16,17,18,19^ Nowadays, use of corticosteroid bursts are considered harmless, an assumption supported by years of clinical data linking exposure duration with toxic effects.^20^ Clinicians currently prescribe short courses of oral corticosteroids to 21% of the general adult population in the US^10^ and up to 17% of the general adult population in France.^21^ Corticosteroid bursts are typically prescribed for treating non–life-threatening conditions, such as upper respiratory tract infections, bronchitis, rashes, and low-back pain.^10,22^ A population-based study by Waljee et al^10^ showed increased rates of adverse events, including sepsis, venous thromboembolism, and fracture, among adults in the US who were treated with oral corticosteroids for fewer than 30 days. A recent longitudinal analysis of 15 million adults in Taiwan by Yao and colleagues^9^ is the first, to our knowledge, to report potential harms of corticosteroid bursts by using a self-controlled case series design. Yao et al^9^ demonstrated increased risks of GI bleeding, sepsis, and heart failure in a general adult population receiving corticosteroid bursts. However, to our knowledge, data regarding the potential harms of short-term oral corticosteroids in children remain limited.

To address this knowledge gap, we used a self-controlled case series design and conducted a nationwide population-based study in Taiwan to evaluate the association of corticosteroid bursts in children with 4 adverse events available in our database, GI bleeding, sepsis, pneumonia, and glaucoma.

## Methods

### Data Source

The National Health Insurance Research Database (NHIRD) comprises medical claims records and prescription data from approximately 23 million individuals covered in the National Health Insurance Program (NHIP) in Taiwan. Approximately 99% of the Taiwanese population has been registered and covered by the NHIP. In this study, we used the deidentified medical claims records and prescription data from the entire NHIRD from January 1, 2013, to December 31, 2017. The institutional review board of the National Health Research Institutes, Taiwan, approved this study protocol, and informed consent was waived because all data were encrypted.

### Study Design and Populations

**Figure 1.**
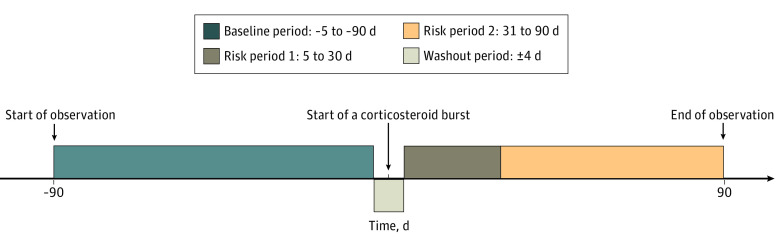
Graphic Presentation of Self-controlled Case Series Design The observation periods are the baseline period and the 2 risk periods.

In this study, we undertook a self-controlled case series to quantify the risks of 4 severe adverse events, GI bleeding, sepsis, pneumonia, and glaucoma, after initiation of a corticosteroid burst. In a self-controlled case series, each participant serves as his or her own control, given unmeasured time-invariant variables automatically controlled for in the succeeding analysis.^23^ The risks of each severe adverse event within the pretreatment period (the reference period defined as 5-90 days prior to initiation of a corticosteroid burst) were compared with the risks within each of 2 posttreatment periods (5-30 days and 31-90 days after initiation of a corticosteroid burst) among participants who received a single corticosteroid burst (Figure 1). We excluded participants who received more than 1 corticosteroid burst during the observation period. We used a conservative approach by including a 4-day washout period. As such, the severe adverse events that occurred during a 4-day window both before and after corticosteroid use were dismissed because the severe adverse events observed among those participants might be due to other factors.

Participants who were enrolled in the NHIP 1 year prior to the study period and during the entire study period were included. Exclusion criteria were (1) 18 years of age or older in 2013; (2) prescription of systemic or topical corticosteroids prior to 2013; (3) diagnosis of GI bleeding, sepsis, pneumonia, or glaucoma prior to 2013; (4) more than 1 corticosteroid burst administered during the observation period; (5) continuous oral corticosteroid prescription for more than 14 days; and (6) congenital anomalies or catastrophic illnesses.

### Exposure and Study Outcomes

Data on the exposure to corticosteroid bursts were obtained from the NHIRD. We summed all successive corticosteroid prescription days since the first corticosteroid prescription through all prescription records in the posttreatment period as “cumulative use days” and identified corticosteroid bursts as continuous use of oral corticosteroids for 14 days or less. To ascertain standardized doses, we converted the investigated corticosteroids into a daily dose based on prednisone equivalent doses (eTable 1 in the Supplement).

Previous studies^3,4,5,6,7,8^ have reported the adverse effects of long-term corticosteroid use on the GI, immune, and ophthalmologic systems; however, to our knowledge, it remains unknown whether corticosteroid bursts are associated with adverse effects on these systems, especially in children. Thus, we chose 4 severe adverse events (GI bleeding, sepsis, pneumonia, and glaucoma) as the outcomes of interest in this study. Episodes of syncope were treated as a negative control outcome. Gastrointestinal bleeding, sepsis, pneumonia, glaucoma, and syncope were defined based on *International Classification of Diseases, Ninth Revision, Clinical Modification* codes for encounters between 2013 and 2015 and *International Statistical Classification of Diseases and Related Health Problems, Tenth Revision, Clinical Modification* codes for 2016 and 2017 (eTable 2 in the Supplement).

### Covariates

The complete list of time-varying covariates included the top 10 diagnosed acute conditions and concomitant medication use for the severe adverse events (eg, nonsteroidal anti-inflammatory drugs [NSAIDs] and proton pump inhibitors for GI bleeding, NSAIDs and systemic immunosuppressive agents for sepsis and pneumonia, and NSAIDs for glaucoma).

### Statistical Analysis

Data were analyzed from January 1 to July 30, 2020. We computed incidence rates per 1000 person-years of the 4 severe adverse events for participants prescribed corticosteroid bursts and participants not prescribed corticosteroids. We calculated incidence rate ratios (IRRs) by comparing the incidence rates of the severe adverse events within each posttreatment period with the incidence rates of the severe adverse events within the reference period. We performed the analyses using conditional fixed-effect Poisson regression. For each severe adverse event and negative control event, stepwise selection was applied to determine the corresponding list of time-varying covariates adjusted in the analytical models (eMethods in the Supplement). To assess the robustness of observed associations, we performed sensitivity analyses to examine (1) the inclusion of participants with prescriptions of topical corticosteroids prior to the study period and (2) the different durations of observation periods, with 180 days as the maximum postexposure time (reference period defined as 5-180 days prior to initiation of a corticosteroid burst and 2 posttreatment periods defined as 5-60 days and 61-180 days after initiation of a corticosteroid burst). We further used E-values to evaluate the association of potential unmeasured confounding.^24^ Subgroup analyses were performed to investigate the number of days of corticosteroid bursts by classifying participants into 2 groups: those who received corticosteroid bursts for less than 7 days vs those who received corticosteroid bursts for 7 days or more. All analyses were performed using SAS, version 9.2 (SAS Institute Inc) and R package, version 3.6.3 (R Group for Statistical Computing).

## Results

### Baseline Characteristics of the Study Participants

The total number of study participants younger than 18 years was 4 542 623. Among those, 1 897 858 (42%) received at least 1 corticosteroid burst during the 5-year study period. In this study, 1 064 587 participants (23%; 544 268 boys [51.1%] and 520 319 girls [48.9%]; mean [SD] age, 9.7 [5.8] years) who received a single corticosteroid burst were included; and 91% had a Charlson Comorbidity Index score of 0. Table 1 shows the baseline characteristics of the study participants who received a single corticosteroid burst or 1 or more corticosteroid bursts during the observational period. Table 1 suggests comparable baseline characteristics between these 2 cohorts and shows the most common indications for use of corticosteroid bursts: acute respiratory tract infections (acute upper respiratory infections [10.2% vs 10.5%], acute bronchitis and bronchiolitis [9.1% vs 10.1%], acute sinusitis [5.6% vs 6.0%], acute tonsillitis [3.4% vs 3.3%], acute laryngitis and tracheitis [3.1% vs 3.0%], and acute nasopharyngitis [2.9% vs 3.2%]) and allergic diseases (urticaria [11.9% vs 11.0%], contact dermatitis and eczema [10.3% vs 9.1%], asthma [5.2% vs 7.1%], and allergic rhinitis [3.4% vs 3.2%]). These indications accounted for 65% of all reasons that corticosteroid bursts were prescribed for participants who received a single corticosteroid burst. The top 5 physician specialties associated with the prescriptions of corticosteroid bursts were pediatrics, dermatology, otolaryngology, family practice, and internal medicine, accounting for 93% of corticosteroid bursts prescribed to participants who received a single corticosteroid burst.

**Table 1.** Characteristics of Children With Corticosteroid Bursts

Characteristic	Children, No. (%)	
	1 Corticosteroid burst (n = 1 064 587)	All corticosteroid burst(s) (n = 1 897 858)
Age, mean (SD), y	9.7 (5.8)	9.5 (5.7)
Female	520 319 (48.9)	897 193 (47.3)
Corticosteroid use		
Daily dose, median (IQR), mg/d	6.00 (1.50-15.00)	8.17 (1.25-15.00)
Duration, median (IQR), d	3.00 (3.00-3.00)	3.00 (3.00-3.00)
Incidence rate per 1000 person-years (95% CI)		
GI bleeding	2.48 (2.44-2.52)	2.54 (2.51-2.57)
Sepsis	0.37 (0.35-0.39)	0.41 (3.96-4.22)
Pneumonia	25.74 (25.59-25.88)	27.86 (27.75-27.98)
Glaucoma	0.62 (0.60-0.64)	0.65 (0.64-0.65)
**Diagnosis of the top 10 acute conditions (*ICD-9-CM* and *ICD-10-CM* codes)**^a^		
Urticaria (708.xx and L50.xxxx)	126 290 (11.9)	20 8957 (11.0)
Contact dermatitis and other eczema (692.xx, L23.xxxx, L24.xxxx, and L25.xxxx)	109 113 (10.3)	172 276 (9.1)
Acute		
Upper respiratory tract infections (465.xx and J06.xxxx)	108 082 (10.2)	198 685 (10.5)
Bronchitis and bronchiolitis (466.xx, J20.xxxx, and J21.xxxx)	96 514 (9.1)	191 826 (10.1)
Sinusitis (461.xx and J01.xxxx)	59 329 (5.6)	113 428 (6.0)
Asthma (493.xx and J45.xxxx)	55 064 (5.2)	13 4658 (7.1)
Allergic rhinitis (477.xx and J30.xxxx)	35 879 (3.4)	60 884 (3.2)
Acute		
Tonsillitis (463.xx and J03.xxxx)	35 683 (3.4)	6640 (3.3)
Laryngitis and tracheitis (464.xx, J04.xxxx, and J05.xxxx)	32 541 (3.1)	56 086 (3.0)
Nasopharyngitis (460.xx and J00.xxxx)	30 847 (2.9)	60 384 (3.2)
**Physician specialty**		
Pediatrics	283 996 (26.7)	558 859 (29.5)
Dermatology	276 286 (26.0)	434 936 (22.9)
Otolaryngology	189 174 (17.8)	336 791 (17.8)
Family practice	159 178 (15.0)	277 690 (14.6)
Internal medicine	78 516 (7.4)	135 956 (7.2)

Abbreviations: GI, gastrointestinal; *ICD-9-CM*, *International Classification of Diseases, Ninth Revision, Clinical Modification*; *ICD-10-CM*, *International Statistical Classification of Diseases, Tenth Revision, Clinical Modification*; IQR, interquartile range.

^a^ *ICD-9-CM* codes were used to define the conditions in 2013 to 2015, and *ICD-10-CM* codes were used to define the conditions in 2016 and 2017 (eTable 2 in the Supplement).

### Incidence Rates of 4 Adverse Events

The incidence rates per 1000 person-years of the 4 severe adverse events (GI bleeding, sepsis, pneumonia, and glaucoma) for participants prescribed a single corticosteroid burst and for participants not prescribed corticosteroids are presented in Table 2. The incidence rates per 1000 person-years of the 4 severe adverse events among participants administered a single corticosteroid burst were greater than those among participants not prescribed corticosteroids. The incidence rate differences per 1000 person-years between the 2 groups were 0.60 (95% CI, 0.55-0.64) for GI bleeding, 0.03 (95% CI, 0.02-0.05) for sepsis, 9.35 (95% CI, 9.19-9.51) for pneumonia, and 0.01 (95% CI, 0.01-0.03) for glaucoma (Table 2).

**Table 2.** Incidence Rates of Gastrointestinal Bleeding, Sepsis, Pneumonia, and Glaucoma in Children With or Without Corticosteroids

Adverse event	Corticosteroid bursts			No corticosteroids			Rate difference per 1000 person-years (95% CI)
	No. of cases	No. of person-years	Incidence rate per 1000 person-years (95% CI)	No. of cases	No. of person-years	Incidence rate per 1000 person-years (95% CI)	
Gastrointestinal bleeding	13 078	5 273 004	2.48 (2.44-2.52)	31 466	16 706 990	1.88 (1.86-1.90)	0.60 (0.55-0.64)
Sepsis	1966	5 306 732	0.37 (0.35-0.39)	5628	16 785 555	0.34 (0.33-0.34)	0.03 (0.02-0.05)
Pneumonia	121 143	4 706 896	25.74 (25.59-25.88)	250 122	15 261 762	16.39 (16.32-16.45)	9.35 (9.19-9.51)
Glaucoma	3279	5 303 115	0.62 (0.60-0.64)	10 200	16 771 943	0.61 (0.60-0.62)	0.01 (0.01-0.03)

### IRRs From Self-controlled Case Series Analysis

**Figure 2.**
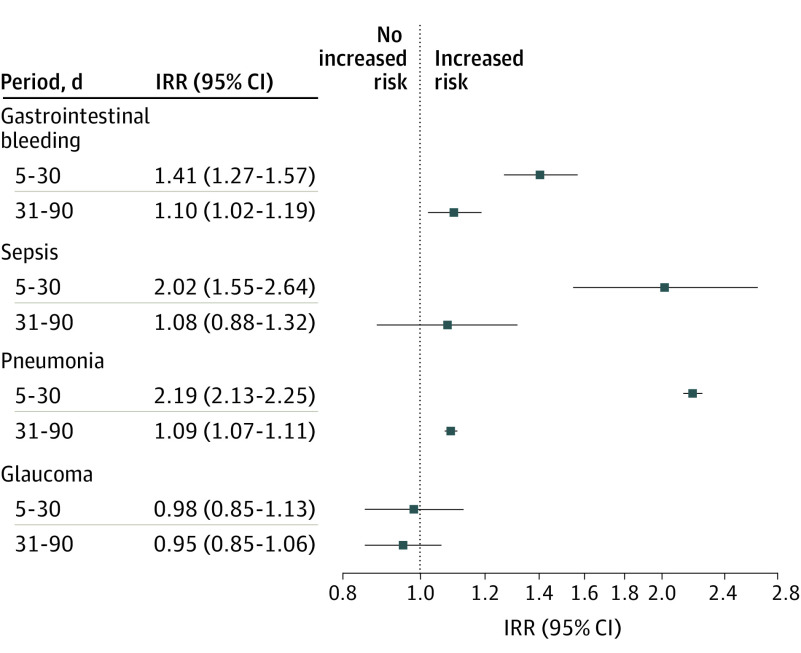
Association Between Exposure to Corticosteroid Bursts and Gastrointestinal Bleeding, Sepsis, Pneumonia, and Glaucoma in Children Incidence rate ratios (IRRs) and corresponding 95% CIs for 4 severe adverse events in 2 posttreatment periods (5-30 days and 31-90 days after initiation of a corticosteroid burst).

Figure 2 shows that the IRRs for GI bleeding and pneumonia across 2 posttreatment periods (5-30 days and 31-90 days after initiating corticosteroid bursts) among participants who received a single corticosteroid burst were significantly higher than the reference period. The IRR for sepsis in the first posttreatment period was significantly greater than the reference period, but not in the second posttreatment period. During the first posttreatment period, the IRR was 1.41 (95% CI, 1.27-1.57) for GI bleeding, 2.02 (95% CI, 1.55-2.64) for sepsis, 2.19 (95% CI, 2.13-2.25) for pneumonia, and 0.98 (95% CI, 0.85-1.13) for glaucoma. During the second posttreatment period, the IRR was 1.10 (95% CI, 1.02-1.19) for GI bleeding, 1.08 (95% CI, 0.88-1.32) for sepsis, 1.09 (95% CI, 1.07-1.11) for pneumonia, and 0.95 (95% CI, 0.85-1.06) for glaucoma. The results in eTable 3 in the Supplement reveal no association of corticosteroid bursts with the risk of syncope, the negative control outcome.

### Sensitivity Analyses

**Figure 3.**
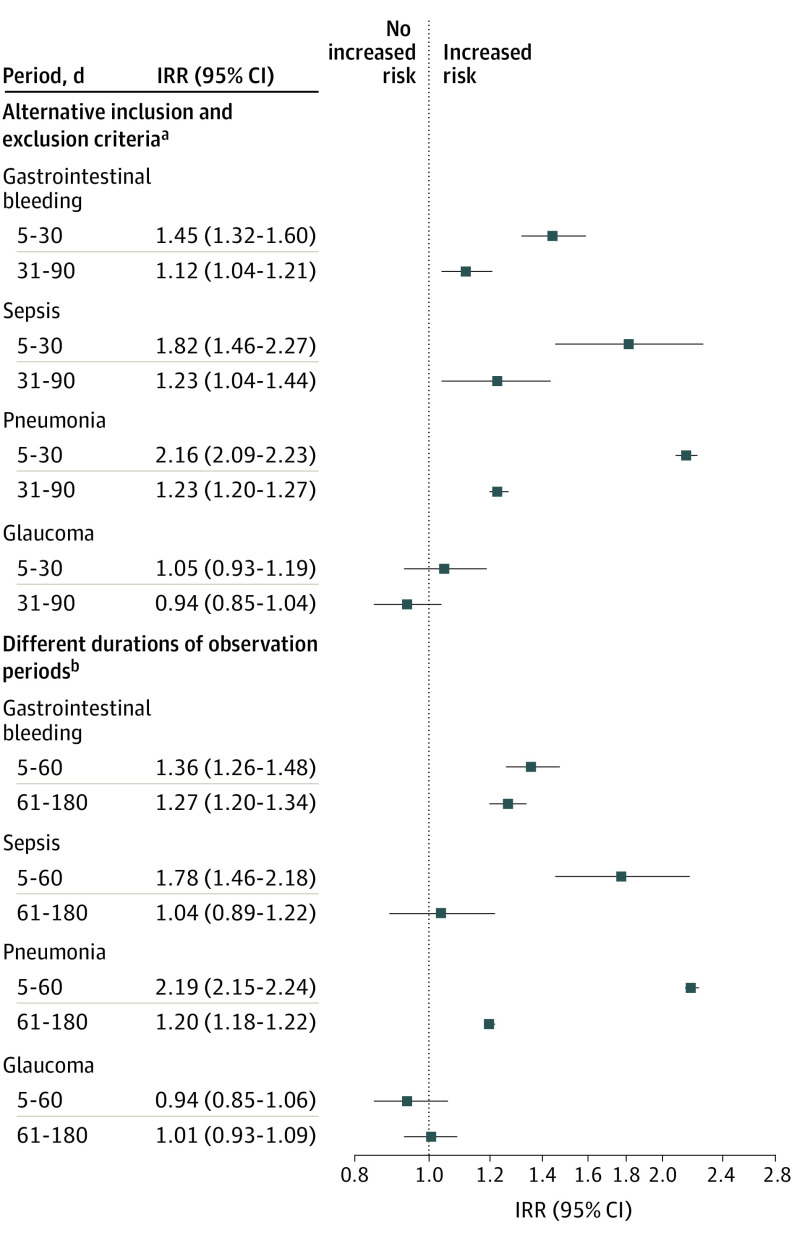
Association Between Exposure to Corticosteroid Bursts and Gastrointestinal Bleeding, Sepsis, Pneumonia, and Glaucoma in Children Based on Alternative Inclusion and Exclusion Criteria and Different Durations of Observation Periods IRR indicates incidence risk ratio. ^a^With inclusion of participants with prescriptions of topical corticosteroids prior to the study period. ^b^With 180 days as the maximum postexposure time (reference period defined as 5-180 days prior to initiation of a corticosteroid burst and 2 posttreatment periods defined as 5-60 days and 61-180 days after initiation of a corticosteroid burst).

Sensitivity analyses were performed to investigate different inclusion and exclusion criteria and different durations of observational periods. The results in Figure 3 were comparable to those in Figure 2, indicating the robustness of the observed associations. We further calculated E-values to evaluate unmeasured confounding for the IRRs reported for the 4 severe adverse events. The E-values ranging from 2.17 to 3.80 for the point estimate of GI bleeding, sepsis, and pneumonia within the first month after corticosteroid initiation suggested no substantial unmeasured confounding (eTable 4 in the Supplement).

### Subgroup Analysis

The results of subgroup analysis in 2 groups are comparable to those reported in the whole study, although some results were not statistically significant in the group of children who used corticosteroids for 7 days or more, probably owing to the decreased sample size (eTable 5 in the Supplement).

## Discussion

In this nationwide population-based study of more than 4 million children, 42% were exposed to at least 1 corticosteroid burst during the 5-year study period. Corticosteroid bursts were typically prescribed for children with acute respiratory tract infections (34%) and allergic diseases (31%). Corticosteroid bursts were significantly associated with a 1.4- to 2.2-fold increase of GI bleeding, sepsis, and pneumonia, but not glaucoma, within the first month after initiation of corticosteroid therapy. This study demonstrates the potential harms of prescribing corticosteroid bursts to children and calls for the prudent use of corticosteroid bursts.

To our knowledge, this is the first and only nationwide, longitudinal, population-based study quantifying the association of corticosteroid bursts with risks of severe adverse events in children. Using a US health care database, Waljee et al^10^ reported increased risks of sepsis, venous thromboembolism, and fracture among adults receiving short-term oral corticosteroids for fewer than 30 days. Using Taiwan’s NHIRD, Yao et al^9^ indicated that corticosteroid bursts in adults are associated with increased risks of GI bleeding, sepsis, and heart failure. Our study extends the risks of severe adverse events associated with corticosteroid bursts from adults to children and provides supportive evidence that treatment with corticosteroid bursts is associated with increased risk of GI bleeding, sepsis, and pneumonia within the first month after initiation of corticosteroid therapy for children.

The findings have several clinical implications. First, sepsis is a rare but potentially life-threatening event. Despite the small observed incidence rate difference in sepsis between children with and children without prescriptions of corticosteroid bursts, corticosteroid bursts were associated with a 2-fold increased risk of sepsis during the first month after starting treatment. Particular caution is therefore needed when administering corticosteroid bursts to children. Second, this study provides evidence that corticosteroid bursts are not innocuous but may pose potentially serious health risks, such as GI bleeding, sepsis, and pneumonia, to children. Clinicians prescribing corticosteroid bursts to children need to weigh the benefits against the risks of severe adverse events. Third, the present findings call for a careful reevaluation regarding the prudent use of corticosteroid bursts in children because of the substantial proportion of children administered corticosteroid bursts in the world.

Among children receiving corticosteroid bursts in our study, 91% had no baseline comorbid condition. Most of the corticosteroid bursts were prescribed for non–life-threatening conditions, including acute respiratory tract infections and allergic diseases. A clinical practice guideline for the management of sore throat indicates a weak recommendation for the use of oral corticosteroids in children aged 5 years or older and in adults.^25^ Dvorin et al^22^ estimated that 11% of adult outpatients with acute respiratory tract infections across the US are treated with systemic corticosteroids. Although some studies showed that corticosteroid bursts mitigated earlier symptoms of acute pharyngitis,^26^ clinical trials showed no efficacy of corticosteroid bursts for acute lower respiratory tract infection^27^ and sinusitis.^28^ Further research is necessary to confirm the high frequency of use of corticosteroid bursts in children with acute respiratory tract infections or other non–life-threatening diseases.

### Limitations

Several limitations deserve mention in our analysis. First, lifestyle factors, including exposure to tobacco smoke and body mass index, are not available in the NHIRD. We therefore used a self-controlled case series design, which is robust to control for time-invariant risk factors. The E-values for GI bleeding, sepsis, and pneumonia suggest that it is very unlikely that unmeasured confounding can explain the observed association of corticosteroid bursts with these severe adverse events. Second, previous studies report that the adverse effects of corticosteroids include the GI, immune, and ophthalmologic systems. Our study assessed the association of corticosteroid bursts with 4 severe adverse events, GI bleeding, sepsis, pneumonia, and glaucoma, among children in Taiwan. Further studies are needed to assess the validity of these findings and other corticosteroid-associated adverse events in other pediatric populations. Third, medication noncompliance is a potential concern for studies based on registry data. However, noncompliance is independent of subsequent severe adverse events and may attenuate the observed risk estimates toward the null. Fourth, we did not explore whether the prescriptions of antibiotics, a broader marker of infection, increased after initiation of the corticosteroid bursts. The prescriptions of antibiotics after the corticosteroid bursts will be worth further investigation. In this study, we were able to control for time-invariant risk factors in individual-level variability but not population-level variability owing to the features of self-controlled case series design.

## Conclusions

This nationwide population-based study demonstrates that oral corticosteroid bursts are commonly prescribed to children for non–life-threatening conditions, including acute respiratory tract infections and allergic diseases. Treatment with corticosteroid bursts is associated with a 1.4- to 2.2-fold increased risk of GI bleeding, sepsis, and pneumonia within the first month after initiation of corticosteroid therapy among children. Clinicians should be aware of these rare but potentially serious adverse events associated with use of corticosteroid bursts for children, particularly during the first month after corticosteroid initiation. These findings provide real-world evidence for clinicians and guideline developers to implement strategies with optimal benefit to risk ratios for preventing avoidable harms from the use of corticosteroid bursts for children.

## Notes

Supplement. eMethods. eTable 1. Equivalent Doses of Corticosteroids Investigated in This Study eTable 2. *ICD-9-CM* and *ICD-10-CM* Codes of Four Adverse Events and Negative Control Outcome **eTable 3.** Incidence Rate Ratios for Syncope (Negative Control Outcome) Associated With Corticosteroid Bursts in Children **eTable 4.** Incidence Rate Ratios and E-Values for Four Adverse Events Associated With Corticosteroid Bursts in Children **eTable 5.** Subgroup Analyses of Children With Various Days of Corticosteroid Use (Less Than 7 Days Versus 7 Days or More)
