# Supplementary material for: Association of Oral Corticosteroid Bursts With Severe Adverse Events in Children
Source: JAMA Pediatr. 2021 Apr 19;175(7):1–7. doi: 10.1001/jamapediatrics.2021.0433 (PMC8056312; doi:10.1001/jamapediatrics.2021.0433)
Supplement: Supplement. — eMethods. eTable 1. Equivalent Doses of Corticosteroids Investigated in This Study eTable 2. ICD-9-CM and ICD-10-CM Codes of Four Adverse Events and Negative Control Outcome eTable 3. Incidence Rate Ratios for Syncope (Negative Control Outcome) Associated With Corticosteroid Bursts in Children eTable 4. Incidence Rate Ratios and E-Values for Four Adverse Events Associated With Corticosteroid Bursts in Children eTable 5. Subgroup Analyses of Children With Various Days of Corticosteroid Use (Less Than 7 Days Versus 7 Days or More) [file jamapediatr-e210433-s001.pdf]

Supplementary Online Content

Yao TC, Wang JY, Chang SM, et al. Association of oral corticosteroid bursts with severe adverse events in children. *JAMA Pediatr.* Published online April 19, 2021. doi:10.1001/jamapediatrics.2021.0433

eMethods.
eTable 1. Equivalent Doses of Corticosteroids Investigated in This Study
eTable 2. *ICD-9-CM* and *ICD-10-CM* Codes of Four Adverse Events and Negative Control Outcome
eTable 3. Incidence Rate Ratios for Syncope (Negative Control Outcome) Associated With Corticosteroid Bursts in Children
eTable 4. Incidence Rate Ratios and E-Values for Four Adverse Events Associated With Corticosteroid Bursts in Children
eTable 5. Subgroup Analyses of Children With Various Days of Corticosteroid Use (Less Than 7 Days Versus 7 Days or More)

This supplementary material has been provided by the authors to give readers additional information about their work.

© 2021 Yao TC et al. *JAMA Pediatrics*.

eMethods.

Time-varying covariates

Time-varying covariates that were included were: acute conditions and concomitant medication use. More specifically, the adjusted time-varying covariates included in the final analytical models are: nonsteroidal anti-inflammatory drugs (NSAIDs), proton-pump inhibitors, contact dermatitis and other eczema, acute bronchitis and bronchiolitis, acute tonsillitis, and allergic rhinitis for **gastrointestinal (GI) bleeding**; NSAIDs, systemic immunosuppressive agents, contact dermatitis and other eczema, acute upper respiratory infections, and acute bronchitis and bronchiolitis for **sepsis**; NSAIDs, systemic immunosuppressive agents, contact dermatitis and other eczema, acute upper respiratory infections, urticaria, acute bronchitis and bronchiolitis, acute sinusitis, acute tonsillitis, acute nasopharyngitis, acute laryngitis and tracheitis, asthma, and allergic rhinitis for **pneumonia**; and NSAIDs, acute laryngitis and tracheitis, acute bronchitis and bronchiolitis, acute nasopharyngitis, acute upper respiratory infections, and allergic rhinitis for **glaucoma**.

© 2021 Yao TC et al. *JAMA Pediatrics*.

eTable 1. Equivalent Doses of Corticosteroids Investigated in This Study

**Corticosteroid**	**Equivalent Dose**
Betamethasone	0.6 mg
Dexamethasone	0.75 mg
Methylprednisolone	4 mg
Triamcinolone	4 mg
Prednisone	5 mg
Prednisolone	5 mg
Hydrocortisone	20 mg
Cortisone	25 mg

© 2021 Yao TC et al. *JAMA Pediatrics*.

eTable 2. *ICD-9-CM* and *ICD-10-CM* Codes of Four Adverse Events and Negative Control Outcome

**Diagnosis**	***ICD-9-CM* Code**	***ICD-10-CM* Code**
**Adverse Event**		
GI Bleeding	530.7x, 531.xx, 531.2x, 531.4x, 531.6x, 532.xx, 532.2x, 532.4x, 532.6x, 533.xx, 533.2x, 533.4x, 533.6x, 534.xx, 534.2x, 534.4x, 534.6x, 569.3x, 535.01, 535.11, 535.21, 535.31, 535.41, 535.51, 535.61, 535.71, 537.83, 537.84, 562.02, 562.03, 562.12, 562.13, 569.85, 578.xx	K22.6xxx, K25.xxxx-K28.xxxx, K29.01xx, K29.21xx, K29.31xx, K29.41xx, K29.51xx, K29.61xx, K29.71xx, K29.81xx, K29.91xx, K31.811x, K31.82xx, K52.81xx, K55.21xx, K56.60xx, K57.01xx, K57.11xx, K57.13xx, K57.21xx, K57.31xx, K57.33xx, K57.81xx, K57.91xx, K57.93xx, K62.5xxx, K92.0xxx-K92.2xxx
Sepsis	038.xx, 785.52, 998.02, 995.90-995.94	A40.xxxx, A41.xxxx, R65.10xx, R65.11xx, R65.20xx, R65.21xx, T81.12XA, T81.12XD, T81.12XS
Pneumonia	481.xx -483.xx, 485.xx -486.xx	J12.xxxx -J18.xxxx
Glaucoma	365.xx	H40.xxxx
**Negative Control Outcome**		
Syncope	780.2x, 992.1x	R55.xxxx

GI = gastrointestinal; ***ICD-9-CM*** = *International Classification of Diseases, Ninth Revision, Clinical Modification*; ***ICD-10-CM*** = *International Classification of Diseases, Tenth Revision, Clinical Modification*.

© 2021 Yao TC et al. *JAMA Pediatrics*.

eTable 3. Incidence Rate Ratios for Syncope (Negative Control Outcome) Associated With Corticosteroid Bursts in Children

**Adverse Event**	**No. of Events**	**Daily Dose (mg/day), Median (IQR)**	**Duration of Using Corticosteroids (days), Median (IQR)**	**5-30 Days***	**31-90 Days***
				**Incidence Rate Ratio (95% CI)**	**Incidence Rate Ratio (95% CI)**
**Syncope**	204	10.00 (1.50-15.00)	3.00 (3.00 -3.00)	1.03 (0.77-1.38)	0.92 (0.74-1.15)

IQR = interquartile range; **CI** = confidence interval.
* Syncope model was adjusted for NSAIDs, acute bronchitis and bronchiolitis, acute laryngitis and tracheitis, asthma, and allergic rhinitis.

© 2021 Yao TC et al. *JAMA Pediatrics*.

eTable 4. Incidence Rate Ratios and E-Values for Four Adverse Events Associated With Corticosteroid Bursts in Children

Adverse Event	No. of Events	Daily Dose (mg/day), Median (IQR)	Duration of Using Corticosteroids (days), Median (IQR)	5-30 Days		31-90 Days	
				**Incidence Rate Ratio (95% CI)**	**E-value (CI[a])**	**Incidence Rate Ratio (95% CI)**	**E-value (CI[a])**
**GI Bleeding**[b]	1,176	10.00 (1.50-15.00)	3.00 (3.00-3.00)	1.41 (1.27-1.57)	2.17 (1.86)	1.10 (1.02-1.19)	1.43 (1.16)
**Sepsis**[c]	230	2.38 (0.60-8.33)	3.00 (3.00-3.00)	2.02 (1.55-2.64)	3.46 (2.47)	1.08 (0.88-1.32)	1.37 (1.00)
**Pneumonia**[d]	19,579	2.03 (0.50-9.33)	3.00 (3.00-3.00)	2.19 (2.13-2.25)	3.80 (3.68)	1.09 (1.07-1.11)	3.21 (1.34)
**Glaucoma**[e]	657	8.00 (1.50-15.00)	3.00 (3.00-3.00)	0.98 (0.85-1.13)	1.16 (1.00)	0.95 (0.85-1.06)	1.29 (1.00)

GI = gastrointestinal; **IQR =** interquartile range; **CI** = confidence interval.

[a] E-values for lower limit of the CI when relative risk>1; E-values for upper limit of the CI when relative risk<1.

[b] GI bleeding model was adjusted for nonsteroidal anti-inflammatory drugs (NSAIDs), proton-pump inhibitors, contact dermatitis and other eczema, acute bronchitis and bronchiolitis, acute tonsillitis, and allergic rhinitis.

[c] Sepsis model was adjusted for NSAIDs, systemic immunosuppressive agents, contact dermatitis and other eczema, acute upper respiratory infections, and acute bronchitis and bronchiolitis.

[d] Pneumonia model was adjusted for NSAIDs, systemic immunosuppressive agents, contact dermatitis and other eczema, acute upper respiratory infections, urticaria, acute bronchitis and bronchiolitis, acute sinusitis, acute tonsillitis, acute nasopharyngitis, acute laryngitis and tracheitis, asthma, and allergic rhinitis.

[e] Glaucoma model was adjusted for NSAIDs, acute laryngitis and tracheitis, acute bronchitis and bronchiolitis, acute nasopharyngitis, acute upper respiratory infections, and allergic rhinitis.

© 2021 Yao TC et al. *JAMA Pediatrics*.

eTable 5. Subgroup Analyses of Children With Various Days of Corticosteroid Use (Less Than 7 Days Versus 7 Days or More)

Adverse Event	5-30 Days	31-90 Days	5-30 Days	31-90 Days
	**Incidence Rate Ratio (95% CI)**	**Incidence Rate Ratio (95% CI)**	**Incidence Rate Ratio (95% CI)**	**Incidence Rate Ratio (95% CI)**
	**Children With Corticosteroid Use for Less Than 7 Days**		**Children With Corticosteroid Use for 7 Days or More**	
**GI Bleeding**[a]	1.40 (1.26-1.56)	1.12 (1.03-1.21)	1.49 (1.08-2.07)	0.89 (0.67-1.18)
**Sepsis**[b]	2.01 (1.53-2.63)	1.07 (0.87-1.32)	1.78 (0.50-6.35)	0.88 (0.34-2.27)
**Pneumonia**[c]	2.22 (2.16-2.28)	1.10 (1.08-1.13)	1.72 (1.52-1.93)	0.85 (0.77-0.94)
**Glaucoma**[d]	0..99 (0.85-1.15)	0.96 (0.85-1.07)	0.94 (0.55-1.63)	0.88 (0.58-1.34)

GI = gastrointestinal; **CI** = confidence interval.

[a] GI bleeding model was adjusted for nonsteroidal anti-inflammatory drugs (NSAIDs), proton-pump inhibitors, contact dermatitis and other eczema, acute bronchitis and bronchiolitis, acute tonsillitis, and allergic rhinitis.

[b] Sepsis model was adjusted for NSAIDs, systemic immunosuppressive agents, contact dermatitis and other eczema, acute upper respiratory infections, and acute bronchitis and bronchiolitis.

[c] Pneumonia model was adjusted for NSAIDs, systemic immunosuppressive agents, contact dermatitis and other eczema, acute upper respiratory infections, urticaria, acute bronchitis and bronchiolitis, acute sinusitis, acute tonsillitis, acute nasopharyngitis, acute laryngitis and tracheitis, asthma, and allergic rhinitis.

[d] Glaucoma model was adjusted for NSAIDs, acute laryngitis and tracheitis, acute bronchitis and bronchiolitis, acute nasopharyngitis, acute upper respiratory infections, and allergic rhinitis.

© 2021 Yao TC et al. *JAMA Pediatrics*.
